# Supplementary material for: C1q/TNF-Related Proteins 1, 6 and 8 Are Involved in Corneal Epithelial Wound Closure by Targeting Relaxin Receptor RXFP1 In Vitro
Source: Int J Mol Sci. 2023 Apr 6;24(7):6839. doi: 10.3390/ijms24076839 (PMC10095411; doi:10.3390/ijms24076839)
Supplement: Supplementary file 1 [file ijms-24-06839-s001.zip › Supplementary Figures S1-S3.pdf]

Supplementary Material

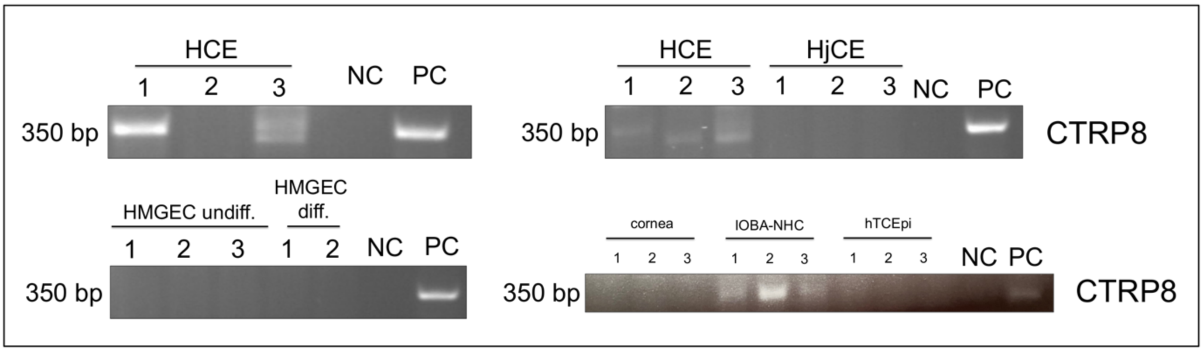

Supplementary Figure S1. CTRP8 gene expression in human tissues and cell lines by RT-PCR. The gene expression is shown by the white bands. Human corneal epithelial cells (HCE), telomerase-immortalized human corneal epithelial cells (hTCEpi), human conjunctival epithelial cells (HCjE), IOBA-normal human conjunctiva (IOBA-NHC), human meibomian gland epithelial cells (HMGEC; differentiated and undifferentiated). Positive control (PC) is testis for CTRP8. Negative control (NC) contains no template cDNA. Pictures are representative for at least at least 3 individual experiments for each tissue and cell line.

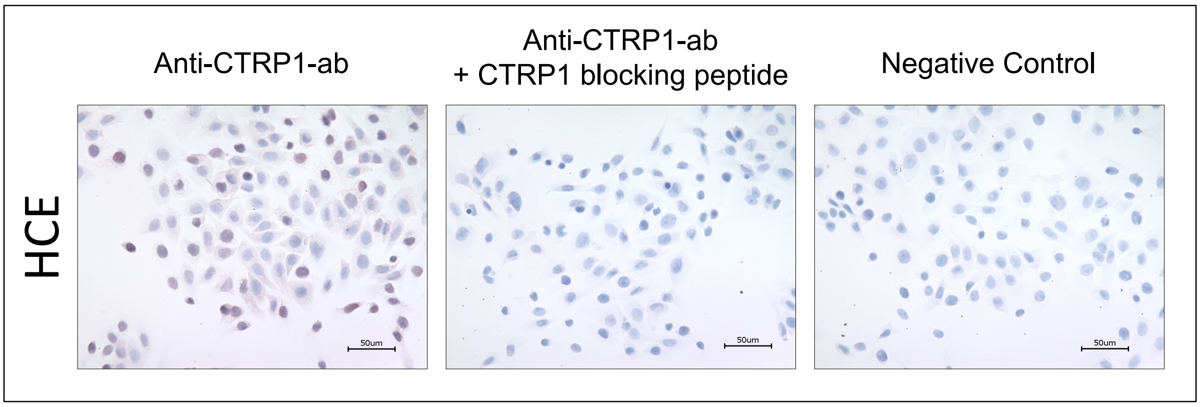

Supplementary Figure S2. CTRP1 antibody (ab) specificity confirmation by antigen pre-adsorption in human corneal epithelial cell line (HCE). The antibody reaction can be seen by the intracellular red reactivity. CTRP1 antibody specificity was confirmed by elimination of antibody reactivity with antigen pre-adsorption with 3 µg CTRP1 blocking peptide/µg antibody.

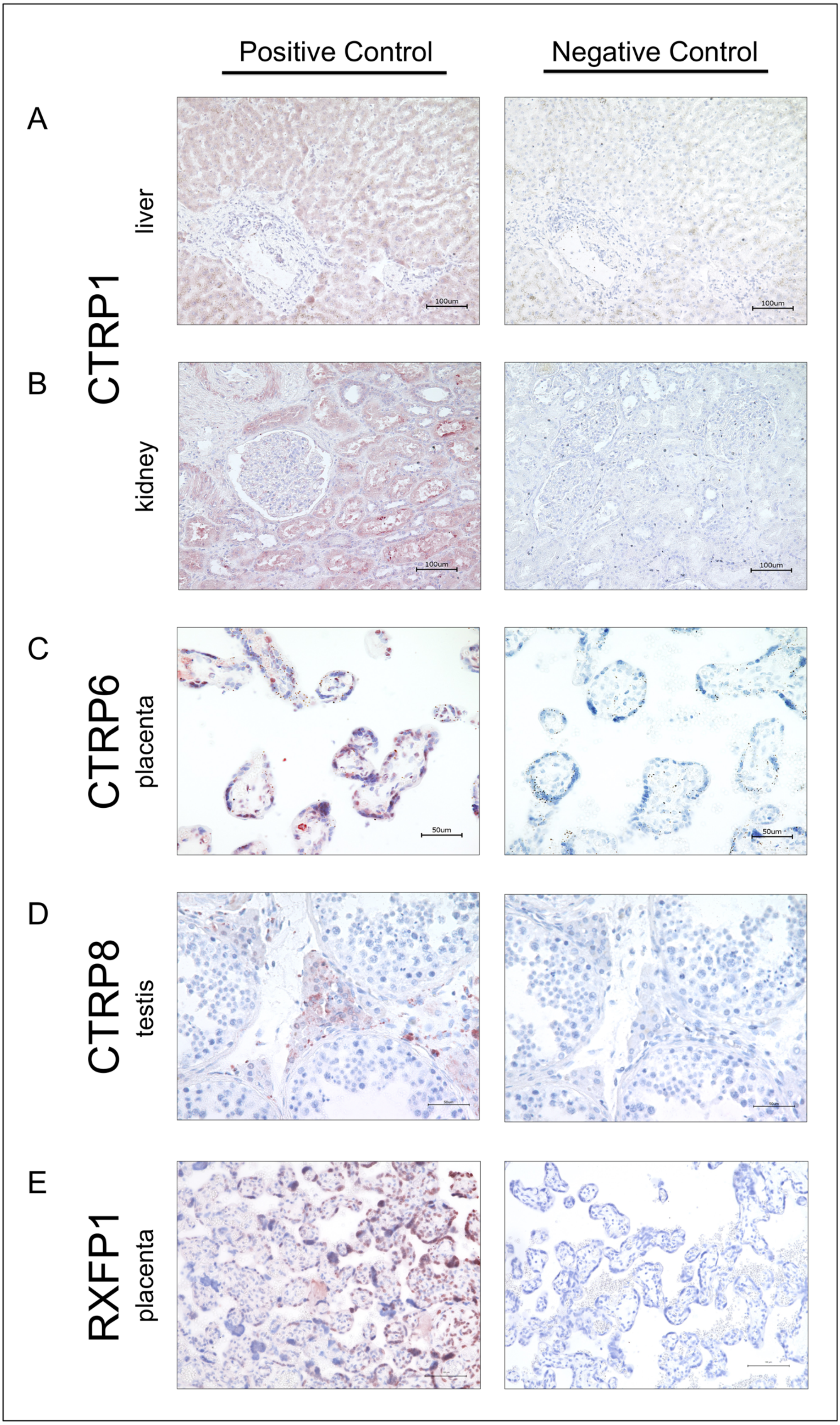

Positive Control
Negative Control
A
B
C
D
E
CTRP1
liver
kidney
CTRP6
placenta
CTRP8
testis
RXFP1
placenta
100um
100um
100um
100um
50um
50um

Supplementary Figure S3. Immunohistochemical detection of CTRP1, CTRP6 and CTRP8 in human tissues (positive controls). The antibody reaction can be seen by the intracellular red reactivity. Positive control for CTRP1 was liver (**A**) and kidney (**B**). Positive control for CTRP6 was placenta (**C**). Positive control for CTRP8 was testis (**D**). Positive control for RXFP1 was placenta (**E**). Negative control sections with non-immune IgG were consistently negative for each of the investigated tissues.
