# Supplementary material for: C1q/TNF-Related Proteins 1, 6 and 8 Are Involved in Corneal Epithelial Wound Closure by Targeting Relaxin Receptor RXFP1 In Vitro
Source: Int J Mol Sci. 2023 Apr 6;24(7):6839. doi: 10.3390/ijms24076839 (PMC10095411; doi:10.3390/ijms24076839)
Supplement: Supplementary file 1 [file ijms-24-06839-s001.zip › Supplementary Tables S1-3.pdf]

Table S1. Relative surface defect area of HCE treated with different concentrations of CTRP1. The table shows the relative surface defect are of HCE treated with either 0 ng/ml (Control), 10 ng/ml or 100 ng/ml CTRP1 at different timepoints. After 12 hours of treatment and for the remainder of the observation period, cells stimulated with 100 ng/ml showed a significantly smaller remaining surface defect area compared to control. There were no significant differences in the surface defect area between HCE treated with 10 ng/ml CTRP1 compared to control and between HCE treated with 100 ng/ml compared to 10 ng/ml CTRP1 (Two-way ANOVA; significance was defined at $p < 0.05$).

	Relative surface defect area (mean ± SEM) [%]			p-value		
**Time [h]**	**0 ng/ml CTRP1 (Control)**	**10 ng/ml CTRP1**	**100 ng/ml CTRP1**	**0 ng/ml vs. 10 ng/ml CTRP1**	**0 ng/ml vs. 100 ng/ml CTRP1**	**10 ng/ml vs. 100 ng/ml CTRP1**
0	100 ± 0	100 ± 0	100 ± 0	-	-	-
6	84.38 ± 1.34	84.15 ± 1.83	80.40 ± 1.56	>0.99	0.12	0.23
12	81.06 ± 1.59	80.00 ± 2.26	73.41 ± 1.56	0.90	0.006	0.06
18	76.06 ± 2.20	75.07 ± 2.55	67.01 ± 2.34	0.94	0.02	0.06
24	70.12 ± 2.91	67.52 ± 3.27	58.29 ± 3.46	0.78	0.03	0.12
36	54.87 ± 3.64	49.14 ± 4.25	37.34 ± 4.43	0.50	0.01	0.13
48	34.11 ± 4.82	31.86 ± 5.18	15.48 ± 4.72	0.93	0.03	0.06

Table S2. Relative surface defect area of HCE treated with different concentrations of CTRP6. The table shows the relative surface defect are of HCE treated with either 0 ng/ml (Control), 10 ng/ml or 100 ng/ml CTRP6 at different timepoints. After 6 hours of treatment and for the remainder of the observation period, cells stimulated with 100 ng/ml showed a significantly smaller remaining surface defect area compared to control. There were no significant differences in the surface defect area between HCE treated with 10 ng/ml CTRP6 compared to control and between HCE treated with 100 ng/ml compared to 10 ng/ml CTRP6 (Two-way ANOVA; significance was defined at $p < 0.05$).

	Relative surface defect area (mean ± SEM) [%]			p-value		
**Time [h]**	**0 ng/ml CTRP6 (Control)**	**10 ng/ml CTRP6**	**100 ng/ml CTRP6**	**0 ng/ml vs. 10 ng/ml CTRP6**	**0 ng/ml vs. 100 ng/ml CTRP6**	**10 ng/ml vs. 100 ng/ml CTRP6**
0	100 ± 0	100 ± 0	100 ± 0	-	-	-
6	80.33 ± 1.08	78.02 ± 1.91	74.92 ± 1.81	0.49	0.04	0.41
12	74.63 ± 1.60	68.62 ± 3.92	63.42 ± 2.61	0.30	0.005	0.46
18	70.22 ± 2.17	61.00 ± 5,73	55.76 ± 3.46	0.27	0.006	0.66
24	62.42 ± 2.67	50.37 ± 7.45	45.33 ± 4.02	0.26	0.006	0.78
36	43.17 ± 3.60	31.75 ± 7.81	20.66 ± 4.43	0.34	0.002	0.39
48	20.32 ± 3.70	17.06 ± 6.47	5.139 ± 2.81	0.87	0.010	0.20

Table S3. Relative surface defect area of HCE treated with different concentrations of CTRP8. The table shows the relative surface defect are of HCE treated with either 0 ng/ml (Control), 10 ng/ml or 100 ng/ml CTRP8 at different timepoints. After 6 hours of, cells stimulated with 100 ng/ml showed a significantly smaller remaining surface defect area compared to control but lost significance at 48 hours. There were no significant differences in the surface defect area between HCE treated with 10 ng/ml CTRP8 compared to control and between HCE treated with 100 ng/ml compared to 10 ng/ml CTRP8 (Two-way ANOVA; significance was defined at $p < 0.05$).

	Relative surface defect area (mean ± SEM) [%]			p-value		
Time [h]	0 ng/ml CTRP8 (Control)	10 ng/ml CTRP8	100 ng/ml CTRP8	0 ng/ml vs. 10 ng/ml CTRP8	0 ng/ml vs. 100 ng/ml CTRP8	10 ng/ml vs. 100 ng/ml CTRP8
0	100 ± 0	100 ± 0	100 ± 0	-	-	-
6	87.51 ± 0.76	85.86 ± 0.84	83.70 ± 1.14	0.27	0.03	0.25
12	87.26 ± 0.78	84.91 ± 1.22	83.08 ± 1.25	0.22	0.03	0.49
18	86.35 ± 0.74	84.62 ± 1.48	81.60 ± 1.29	0.49	0.01	0.24
24	85.00 ± 0.96	82.55 ± 1.43	80.25 ± 1.31	0.29	0.02	0.41
36	81.37 ± 1.45	77.33 ± 2.20	75.02 ± 1.82	0.25	0.03	0.64
48	74.15 ± 2.18	70.88 ± 2.87	68.69 ± 2.43	0.58	0.19	0.79
